# Critically synchronized brain waves form an effective, robust and flexible basis for human memory and learning

**DOI:** 10.1038/s41598-023-31365-6

**Published:** 2023-03-16

**Authors:** Vitaly L. Galinsky, Lawrence R. Frank

**Affiliations:** 1 Center for Scientific Computation in Imaging, University of California at San Diego, La Jolla, CA 92037-0854 USA; 2 Center for Functional MRI, University of California at San Diego, La Jolla, CA 92037-0677 USA

**Keywords:** Neuroscience, Dynamical systems, Learning algorithms, Network models

## Abstract

The effectiveness, robustness, and flexibility of memory and learning constitute the very essence of human natural intelligence, cognition, and consciousness. However, currently accepted views on these subjects have, to date, been put forth without any basis on a true physical theory of how the brain communicates internally via its electrical signals. This lack of a solid theoretical framework has implications not only for our understanding of how the brain works, but also for wide range of computational models developed from the standard orthodox view of brain neuronal organization and brain network derived functioning based on the Hodgkin–Huxley ad-hoc circuit analogies that have produced a multitude of Artificial, Recurrent, Convolution, Spiking, etc., Neural Networks (ARCSe NNs) that have in turn led to the standard algorithms that form the basis of artificial intelligence (AI) and machine learning (ML) methods. Our hypothesis, based upon our recently developed physical model of weakly evanescent brain wave propagation (WETCOW) is that, contrary to the current orthodox model that brain neurons just integrate and fire under accompaniment of slow leaking, they can instead perform much more sophisticated tasks of efficient coherent synchronization/desynchronization guided by the collective influence of propagating nonlinear near critical brain waves, the waves that currently assumed to be nothing but inconsequential subthreshold noise. In this paper we highlight the learning and memory capabilities of our WETCOW framework and then apply it to the specific application of AI/ML and Neural Networks. We demonstrate that the learning inspired by these critically synchronized brain waves is shallow, yet its timing and accuracy outperforms deep ARCSe counterparts on standard test datasets. These results have implications for both our understanding of brain function and for the wide range of AI/ML applications.

## Introduction

The mechanisms of human memory remains one of the great unsolved mysteries in modern science. As a critical component of human learning, the lack of a coherent theory of memory has far-reaching implications for our understanding of cognition as well. Recent advances in experimental neuroscience and neuroimaging have highlighted the importance of considering the interactions of the wide-range of spatial and temporal scales at play in brain function, from the microscales of subcellular dendrites, synapses, axons, somata, to the mesoscales of the interacting networks of neural circuitry, the macroscales of brain-wide circuits. Current theories derived from these experimental data suggest that ability of humans to learn and adapt to ever-changing external stimuli is predicated on the development of complex, adaptable, efficient, and robust circuits, networks, and architectures derived from a flexible arrangements among the variety of neuronal and non-neuronal cell types in the brain. A viable theory of memory and learning must therefore be predicated on a physical model capable of producing multiscale spatiotemporal phenomena consistent with observed data.

At the heart of all current models for brain electrical activity is the neuron spiking model formulated by Hodgkin and Huxley (HH)^1^ that has provided quantitative descriptions of Na^+^/K^+^ fluxes, voltage- and time-dependent conductance changes, the waveforms of action potentials, and the conduction of action potentials along nerve fibers^2^. Unfortunately, although the HH model has been useful in fitting multiparametric set of equations to local membrane measurements, the model has been of limited utility in deciphering complex functions arising in interconnected networks of brain neurons^3^. From a practical standpoint, the original HH model is too complicated to to describe even relatively small networks^4–6^. This has resulted in the development of optimization techniques^7–10^ based on a much reduced model of a leaky integrate-and-fire (LIF) neuron that is simple enough for use in neural networks, as it replaces all these multiple gates, currents, channels and thresholds with just a single threshold and time constant. A majority of spiking neural network (SNN) models use this simplistic LIF neuron for the so called “deep learning”^11–14^ claiming that this is inspired by brain functioning. While multiple LIF models are used for image classification on large datasets^15–19^, most applications of SNNs are still limited to less complex datasets, due to the complex dynamics of even the oversimplified LIF model and non-differentiable operations of LIF spiking neurons. Some remarkable studies have applied SNNs for object detection tasks^20–22^. Spike based methods were also used for object tracking^23–26^. A research is booming in using LIF spiking networks for online learning^27^, braille letter reading^28^, different neuromorphic synaptic devices^29^ for detection and classification of biological problems^30–36^. Significant research is focused on making human-level control^37^, optimizing back-propagation algorithms for spiking networks^38–40^, as well as penetrating much deeper into ARCSes core^41–44^ with smaller number of time steps^41^, using an event-driven paradigm^36, 40, 45, 46^, applying batch normalization^47^, scatter-and-gather optimizations^48^, supervised plasticity^49^, time-step binary maps^50^, and using transfer learning algorithms^51^. In concert with this broad range of software applications, there is a huge amount of research directed at developing and using these LIF SNN in embedded applications with the help of the *neuromorphic hardware*^52–57^, the generic name given to hardware that is nominally based on, or inspired by, the structure and function of the human brain. However, while the LIF model is widely accepted and ubiquitous in neuroscience, it is nevertheless problematic in that it does not generate any spikes per se.

A single LIF neuron can formally be described in differential form as

1 $$\begin{aligned} \tau _{m}\frac{\partial U}{\partial t}&= -(U-U_{rest}) + R I, \end{aligned}$$

where *U*(*t*) is the membrane potential, $U_{rest}$ is the resting potential, $\tau _m$ is the membrane time constant, *R* is the input resistance, and *I*(*t*) is the input current^58^. It is important to note that Eq. (1) does *not* describe actual spiking. Rather, it integrates the input current *I*(*t*) in the presence of an input membrane voltage *U*(*t*). In the absence of the current *I*(*t*), the membrane voltage rapidly (exponentially) decays with time constant $\tau _m$ to its resting potential $U_{rest}$. In this sense the integration is “leaky”. There is no structure in this equation that even approximates a system resonance that might be described as “spiking”. Moreover, both the decay constant $\tau _m$ and the resting potential $U_{rest}$ are not only unknowns, but assumed constant, and therefore significant oversimplifications of the actual complex tissue environment.

It is a curious development in the history of neuroscience that the mismatch between the observed spiking behavior of neurons and a model of the system that is incapable of producing spiking was met not with a reformulation to a more physically realistic model, but instead with what can only be described as an ad-hoc patchwork fix: the introduction of a “firing threshold” $\Theta$ that defines when a neuron finally stops integrating the input, resulting in a large action potential almost magically shared with its neighboring neurons, after which the membrane voltage *U* is reset by hand back to the resting potential $U_{rest}$. Adding these conditions results in (1) being only capable of describing the dynamics that happen when the membrane potential *U* is below this spiking ruler threshold $\Theta$. It is important to recognize that this description of the “sub-threshold” dynamics of the membrane potential until it has reached its firing threshold describes a situation where neighboring neurons are *not effected* by what is essentially a description of sub-threshold noise.

In short, the physical situation described by (1) is contradictory to many careful neuroscience experiments that show, for example, that (1) the neuron is anisotropically activated following the origin of the arriving signals to the membrane; (2) a single neuron’s spike waveform typically varies as a function of the stimulation location; (3) spatial summation is absent for extracellular stimulations from different directions; (4) spatial summation and subtraction are not achieved when combining intra- and extra-cellular stimulations, as well as for nonlocal time interference^59^. Such observation have lead to calls “to re-examine neuronal functionalities beyond the traditional framework”^59^.

Such a re-examination has been underway in our lab, where we have developed a physics based model of brain electrical activity. We have demonstrated that in the inhomogeneous anisotropic brain tissue system, the underlying dynamics is not necessarily restricted by reaction–diffusion type only. The recently developed theory of weakly evanescent brain waves (WETCOW)^60–62^ shows from a physical point of view that propagation of electromagnetic fields through the highly complex geometry of inhomogeneous and anisotropic domain of real brain tissues can also happen in a wave-like form . This wave-like propagation agrees well with the results of the above neuronal experiments^59^ as well as in general explains the broad range of observed seemingly disparate brain spatiotemporal characteristics. The theory produces a set of nonlinear equations for both the temporal and spatial evolution of brain wave modes that include all possible nonlinear interaction between propagating modes at multiple spatial and temporal scales and degrees of nonlinearity. The theory bridges the gap between the two seemingly unrelated spiking and wave ‘camps’ as the generated wave dynamics includes the complete spectra of brain activity ranging from incoherent asynchronous spatial or temporal spiking events, to coherent wave-like propagating modes in either temporal or spatial domains, to collectively synchronized spiking of multiple temporal or spatial modes.

In this paper we highlight some particular aspects of the WETCOW theory directly related to biological learning through wave dynamics, and demonstrate how these principles can not only augment our understanding of cognition, but provide the basis for a novel class of engineering analogs for both software and hardware learning systems that can operate with the extreme energy and data efficiency characteristics of biological systems that facilitate adaptive resilience in dynamic environments.

We would like to emphasize that a major motivation for our work is the recognition that there has been a rapidly growing focus in the research community in recent years on theories of memory, learning, and consciousness rely on networks of HH (LIF) neurons as biological and/or physical basis^63^. Every single neuron in this case is assumed to be an element (or a node) with fixed properties that isotropically collects input and fires when enough has been collected. The learning algorithms are then discussed as processes that update network properties, e.g., connection strength between those fixed nodes through plasticity^64^, or number of participating fixed neuron nodes in the network through birth and recruitment of new neuron nodes^65^, etc. In our paper, we focus on different aspect of network functioning—we assume that network is formed not by fixed nodes (neurons) but by a flexible pathways encompassing propagating waves, or wave packets, or wave modes. Formally those wave modes play in any network of wave modes the same role as single HH (LIF) node in network of neurons, therefore, we often interchangeably use and substitute ‘wave mode’ for ‘network node’. But, as any single neuron may encounter multiple wave modes arriving from any other neuron, and synchronization with or without spiking will manifest as something that looks like anisotropic activation depending on the origin of the arriving signals^59^, our wave network paradigm is capable of characterizing much more complex and subtle coherent brain activity and thus shows more feature-rich possibilities for “learning” and memory formation.

The test examples based on our WETCOW inspired algorithms show excellent performance and accuracy and can be expected to be resilient to catastrophic forgetting, will demonstrate real-time sensing, learning, decision making, and prediction. Due to very efficient, fast, robust and very precise spike synchronization, the WETCOW based algorithms are able to respond to novel, uncertain, and rapidly changing conditions in real-time, and will enable appropriate decisions based on small amounts of data over short time horizons. These algorithms can include uncertainty quantification for data of high sparsity, large size, mixed modalities, and diverse distributions, and will be pushing the bounds on out-of-distribution generalization.

The WETCOW model is supposed to capture several different memory phenomena. In a non-mathematical way it can be described as

- Critical encoding–the WETCOW model shows how independent oscillators in a heterogeneous network with different parameters form a coherent state (memory) as a result of critical synchronization.
- Synchronization speed—the WETCOW model shows that due to coordinated work of amplitude-phase coupling this synchronization process is significantly more fast than memory formation in the spiking network of integrate-and-fire neurons.
- Storage capacity—the WETCOW model shows that a coherent memory state with predicted encoding parameters can be formed with as low as two nodes, thus potentially allows for significant increase of memory capacity comparing to the traditional spiking paradigm.
- Learning efficiency—the WETCOW model shows that processing of a new information by a mean of synchronization of network parameters in a near critical range allows a natural development of continuous learner-type memory representative of human knowledge processing.
- Memory robustness—the WETCOW model shows that memory state formed in non-planar critically synchronized network potentially more stable, continuous learning prone, and resilient to catastrophic forgetting.

The experiments presented below are clearly beyond human training capacity, but nevertheless represent a very good set of preliminary stress tests to provide support for all our claims, from Critical Encoding to Memory Robustness. The demonstration that back propagation step is not generally required for very good performance of continues learning as well as small footprint of nodes and input data involved in memory formation are representative of human few-shot learning^66–69^ as well as relevant to the larger issues introduced in our paper.

## Weakly evanescent brain waves

A set of derivations that lead to the WETCOW description was presented in details in^60–62^ and is based on considerations that follow from the most general form of brain electromagnetic activity expressed by Maxwell equations in inhomogeneous and anisotropic medium^70–72^

$$\begin{aligned} \nabla \cdot \varvec{D}&= \rho ,\quad \nabla \times \varvec{H} = \varvec{J} + \frac{\partial \varvec{D}}{\partial t}\quad \Rightarrow \quad \frac{\partial \rho }{\partial t} + \varvec{\nabla }\cdot \varvec{J} = 0. \end{aligned}$$

Using the electrostatic potential $\varvec{E}=-\nabla \Psi$, Ohm’s law $\varvec{J}=\varvec{\sigma }\cdot \varvec{E}$ (where $\varvec{\sigma }\equiv \{\sigma _{ij}\}$ is an anisotropic conductivity tensor), a linear electrostatic property for brain tissue $\varvec{D}=\varepsilon \varvec{E}$, assuming that the scalar permittivity $\varepsilon$ is a “good” function (i.e. it does not go to zero or infinity everywhere) and taking the change of variables $\partial x \rightarrow \varepsilon \partial x^\prime$, the charge continuity equation for the spatial–temporal evolution of the potential $\Psi$ can be written in terms of a permittivity scaled conductivity tensor $\varvec{\Sigma }=\{\sigma _{ij}/\varepsilon \}$ as

2 $$\begin{aligned} \frac{\partial }{\partial t} \left( \nabla ^2 \Psi \right)&= -\varvec{\nabla }\cdot \varvec{\Sigma }\cdot \nabla \Psi + {\mathcal {F}}, \end{aligned}$$

where we have included a possible external source (or forcing) term ${\mathcal {F}}$. For brain fiber tissues the conductivity tensor $\varvec{\Sigma }$ might have significantly larger values along the fiber direction than across them. The charge continuity without forcing i.e., (${\mathcal {F}}=0$) can be written in tensor notation as

3 $$\begin{aligned} \partial _t \partial _i^2 \Psi&+ \Sigma _{ij}\partial _i\partial _j \Psi + \left( \partial _i\Sigma _{ij}\right) \left( \partial _j\Psi \right) =0, \end{aligned}$$

where repeating indices denote summation. Simple linear wave analysis, i.e. substitution of $\Psi \sim \exp {(-i(\varvec{k}\cdot \varvec{r}-\Omega t)})$, where $\varvec{k}$ is the wavenumber, $\varvec{r}$ is the coordinate, $\Omega$ is the frequency and *t* is the time, gives the following complex dispersion relation:

4 $$\begin{aligned} D(\Omega ,\varvec{k}) = -i\Omega k_i^2 - \Sigma _{ij}k_i k_j - i \partial _i\Sigma _{ij} k_j = 0, \end{aligned}$$

which is composed of the real and imaginary components:

5 $$\begin{aligned} \gamma \equiv \Im \Omega = \Sigma _{ij}\frac{k_i k_j}{k^2} \qquad \omega \equiv \Re \Omega =-\frac{\partial _i\Sigma _{ij} k_j}{k^2} \end{aligned}$$

Although in this general form the electrostatic potential $\Psi$, as well as the dispersion relation $D(\Omega ,\varvec{k})$, describe three dimensional wave propagation, we have shown^60, 61^ that in anisotropic and inhomogeneous media some directions of wave propagation are more equal than others with preferred directions determined by the complex interplay of the anisotropy tensor and the inhomogeneity gradient. While this is of significant practical importance, in particular because the anisotropy and inhomogeneity can be directly estimated from non-invasive methods, for the sake of clarity we focus here on the one dimensional scalar expressions for spatial variables x and k that can be easily generalized for the multi dimensional wave propagation as well.

Based on our nonlinear Hamiltonian formulation of the WETCOW theory^62^, there exists an anharmonic wave mode

6 $$\begin{aligned} H^s(a,a^\dag )&= \Gamma a a^\dag \! + a a^\dag \! \left[ \beta _a a + \beta _{a^\dag } a^\dag \! - 2\alpha \left( a a^\dag \right) ^{1/2}\right] \end{aligned}$$

where *a* is a complex wave amplitude and $a^\dag$ is its conjugate. The amplitude *a* denotes either temporal $a_k(t)$ or spatial $a_\omega (x)$ wave mode amplitudes that are related to the spatiotemporal wave field $\Psi (x,t)$ through a Fourier integral expansions

7 $$\begin{aligned}&a_k(t)=\frac{1}{2\pi } \int \limits _{-\infty }^{\infty } \Psi (x,t) e^{-i\left( k x + \omega _kt\right) }dx, \end{aligned}$$

8 $$\begin{aligned}&a_\omega (x) = \frac{1}{2\pi }\int \limits _{-\infty }^{\infty } \Psi (x,t) e^{-i\left( k_\omega x + \omega t\right) }dt, \end{aligned}$$

where for the sake of clarity we use one dimensional scalar expressions for spatial variables *x* and *k*, but it can be easily generalized for the multi dimensional wave propagation as well. The frequency $\omega$ and the wave number *k* of the wave modes satisfy the dispersion relation $D(\omega ,k)=0$, and $\omega _k$ and $k_\omega$ denote the frequency and the wave number roots of the dispersion relation (the structure of the dispersion relation and its connection to the brain tissue properties has been discussed in^60^).

The first term $\Gamma a a^\dag$ in (6) denotes the harmonic (quadratic) part of the Hamiltonian with either the complex valued frequency $\Gamma =i\omega +\gamma$ or wave number $\Gamma =i k +\lambda$ that both include a pure oscillatory parts ($\omega$ or *k*) and possible weakly excitation or damping rates, either temporal $\gamma$ or spatial $\lambda$. The second anharmonic term is cubic in the lowest order of nonlinearity and describes the interactions between various propagating and nonpropagating wave modes, where $\alpha$, $\beta _a$ and $\beta _{a^\dag }$ are the complex valued strengths of those different nonlinear processes. This theory can be extended to a network of interacting wave modes of the form (6) which can be described by a network Hamiltonian form that describes discrete spectrum of those multiple wave modes as^62^

9 $$\begin{aligned} H(\varvec{a},\varvec{a}^\dag )\!&=\! \sum _n\! \left[ \! H^s(a_n,a_n^\dag )\! +\! \sum _{m\ne n}\! \left( a_n r_{nm} a_m^\dag + a_n^\dag r^*_{nm} a_m \right) \!\right] \end{aligned}$$

where the single mode amplitude $a_n$ again denotes either $a_k$ or $a_\omega$, $\varvec{a} \equiv \{a_n\}$ and $r_{nm}=w_{nm}e^{i\delta _{nm}}$ is the complex network adjacency matrix with $w_{nm}$ providing the coupling power and $\delta _{nm}$ taking into account any possible differences in phase between network nodes. This description includes both amplitude $\Re (a)$ and phase $\Im (a)$ mode coupling and as shown in^62^ allows for significantly unique synchronization behavior different from both phase coupled Kuramoto oscillator networks^73–75^ and from networks of amplitude coupled integrate-and-fire neuronal units^58, 76, 77^.

An equation for the nonlinear oscillatory amplitude *a* then can be expressed as a derivative of the Hamiltonian form

10 $$\begin{aligned} \frac{d {a}}{d {t}}=\frac{\partial {H^s}}{\partial {a^\dag }}\equiv \Gamma a + \beta _{a^\dag } a a^\dag + \beta _a a^2 - \alpha a (a a^\dag )^{1/2}, \end{aligned}$$

after removing the constants with a substitution of $\beta _{a^\dag }=1/2 {\tilde{\beta }}_{a^\dag }$ and $\alpha =1/3{\tilde{\alpha }}$ and dropping the tilde. We note that although (10) is an equation for the temporal evolution, the spatial evolution of the mode amplitudes $a_\omega (x)$ can be described by a similar equation substituting temporal variables by their spatial counterparts, i.e., $(t,\omega ,\gamma ) \rightarrow (x,k,\lambda )$.

Splitting (10) into an amplitude/phase pair of equations using $a=Ae^{i\phi }$ and making some rearrangements these equations can be rewritten as^70–72^

11 $$\begin{aligned} \frac{d {A}}{d {\tau }}&=\gamma A + A^2\left[ w^a \cos {(\phi -\psi )}- \alpha \right] , \end{aligned}$$

12 $$\begin{aligned} \frac{d {\phi }}{d {\tau }}&=\omega + A w^\phi \cos {\phi }, \end{aligned}$$

where $\psi$, $w^a$ and $w^\phi$ some model constants.

## Single mode firing rate

The effective period of spiking ${T}_s$ (or its inverse—either the firing rate 1/${T}_s$ or the effective firing frequency $\omega _s = 2\pi /{T}_s$) was estimated in^70–72^ as

13 $$\begin{aligned} T_s&= \frac{2\pi }{\omega \sqrt{1-{\gamma ^2}/{\gamma _c^2}}} = \frac{2\pi }{\omega \sqrt{1 - \omega _c^2/\omega ^2}}, \end{aligned}$$

14 $$\begin{aligned} \omega _s&= \omega \sqrt{1-\gamma ^2/\gamma _c^2}= \sqrt{\omega ^2-\omega _c^2}, \end{aligned}$$

where the critical frequency $\omega _c$ or the critical growth rate $\gamma _c$ can be expressed as

15 $$\begin{aligned} \omega _c =\gamma w,\qquad \gamma _c =\frac{\omega }{w}, \end{aligned}$$

where

16 $$\begin{aligned} w = \frac{w^\phi \cos {\phi ^c}}{\alpha +w^a\cos {(\phi ^c+\psi )}}, \end{aligned}$$

and

17 $$\begin{aligned} \phi ^c = \arctan \left[ \frac{w^a\sin {\psi }}{\sqrt{\alpha ^2-(w^a\sin {\psi })^2}}\right] . \end{aligned}$$

**Figure 1:**
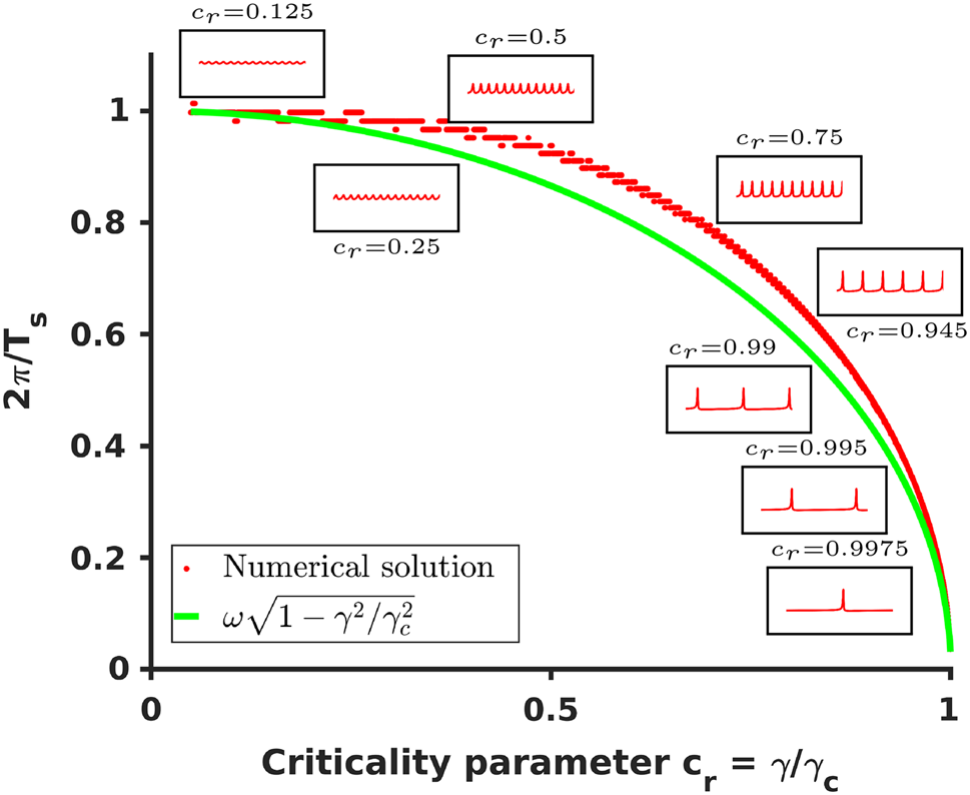
Plot of the analytical expression (14) for the effective spiking frequency $\omega _s=2\pi /T_s$ (green) and the frequency estimated from numerical solutions of (11) and (12) (red) with several inserts showing the numerical solution with indicated value of the criticality parameter $c_r = \gamma /\gamma _c$ (detailed plots of numerical solutions used for generating inserts are included in “Appendix”). In the numerical solution only $\gamma$ was varied and the remaining parameters were the same as parameters reported in^62^.

Figure 1 compares the single node results (13) to (15) with peak-to-peak period/frequency estimates from direct simulations of the system (11) to (12). Several inserts show shapes of numerical solution generated at the correspondent level of criticality $c_r$

18 $$\begin{aligned} c_r = \frac{\gamma }{\gamma _c}&= w \frac{\gamma }{\omega }. \end{aligned}$$

The above analytically derived single node results (13) to (15) can be directly used to estimate firing of interconnected networks as they express the rate of spiking as a function of a distance from criticality, and the criticality value can be in turn expressed through other system parameters.

A set of coupled equations for a network of multiple modes can be derived similarly to single mode set (11) and (12) by taking a derivative of the network Hamiltonian form (9) and appropriately changing variables. That gives for the amplitude $A_i$ and the phase $\phi _i$ a set of coupled equations

19 $$\begin{aligned} \frac{d {A_i}}{d {t}}&= \gamma _i A_i + A_i^2 \left( w^a_i\cos {(\phi _i-\psi _i)} - \alpha _i\right) + \sum _{j\ne i} w_{ij}A_j\cos (\phi _{j} - \phi _{i} - \delta _{ij}), \end{aligned}$$

20 $$\begin{aligned} A_i\frac{d {\phi _i}}{d {t}}&=\omega _i A_i + A_i^2 w^\phi _i \cos \phi _i + \sum _{j\ne i} w_{ij}A_j\sin (\phi _{j} - \phi _{i} - \delta _{ij}). \end{aligned}$$

In the small (and constant) amplitude limit ($A_i=$ const) this set of equations turns into a set of phase coupled harmonic oscillators with a familiar $\sin (\phi _j-\phi _i \cdots )$ form of phase coupling. But in its general form (19) and (20) include also phase dependent coupling of amplitudes ($\cos (\phi _j-\phi _i \cdots )$) that dynamically defines if the input from *j* to *i* will either play excitatory ($|\phi _j-\phi _i +\cdots |<\pi /2$) or inhibitory ($|\phi _j-\phi _i +\cdots |>\pi /2$) roles (this is in addition to any phase shift introduced by the static network attributed phase delay factors $\delta _{ij}$).

## Synchronized network memory of a single node sensory response

Let us start with a single unconnected mode that is excited by a sensory input. Based on the strength of excitation the mode can be in any of the states shown in Fig. 1, with activity ranging from small amplitude oscillations in linear regime, to nonlinear anharmonic oscillations, to spiking with different rates (or effective frequencies) in sub-critical regime, to a single spike-like transition following by silence in supercritical range of excitation. The type of activity is determined by the criticality parameter $c_r=(\gamma _0+\gamma _i)/\gamma _c$ where $\gamma _c$ depends on the parameters of the system (15) and $\gamma _0$ determines the level of sensory input and $\gamma _i$ is the level of background activation (either excitation or inhibition). Hence, for any arbitrary *i*th mode

21 $$\begin{aligned} \frac{d {A_i}}{d {t}}&= (\gamma _0 + \gamma _i) A_i + A_i^2 \left( w^a_i\cos {(\phi _i-\psi _i)} - \alpha _i\right) \end{aligned}$$

22 $$\begin{aligned} \frac{d {\phi _i}}{d {t}}&=\omega _i+ A_i w^\phi _i \cos \phi _i. \end{aligned}$$

As a result, the mode *i* will show nonlinear oscillation with an effective frequency $\omega _s$

23 $$\begin{aligned} \omega _s&= \sqrt{\omega _i^2 - (\gamma _0+\gamma _i)^2 w_i^2}, \end{aligned}$$

24 $$\begin{aligned} w_i&= \frac{w^\phi _i\cos {\phi _i^c}}{\alpha _i+w^a_i\cos {(\phi _i^c+\psi _i)}}, \end{aligned}$$

25 $$\begin{aligned} \phi ^c_i&= \arctan \left[ \frac{w^a_i\sin {\psi _i}}{\sqrt{\alpha _i^2-(w^a_i\sin {\psi _i})^2}}\right] . \end{aligned}$$

Next we assume that instead of a single mode we have some network of modes described by (11) and (12) where the sensory excitation is absent ($\gamma _0 = 0$) and for simplicity we first assume that all the parameters ($\gamma _i$, $\omega _i$,$\alpha _i$,$\psi _i$,$w^a_i$, and $w^\phi _i$) are the same for all modes and only the coupling parameters $w_{ij}$ and $\delta _{ij}$ can vary. The mean excitation level for the network $\gamma _1 \equiv \gamma _i$ ($i=1\ldots N$) determines the type of activity the unconnected modes would be operating and it may be in any of the liner, nonlinear, sub-critical or supercritical range. Of course, the activity of individual nodes in network (11) and (12) depends on the details of coupling (parameters $w_{ij}$ and $\delta _{ij}$) and can be very complex. Nevertheless, at it was shown in^62^, one of the features of the phase–amplitude coupled system (11) and (12), that distinguishes it both from networks of phase coupled Kuramoto oscillators and from networks of amplitude coupled integrate and fire neurons (or actually from any networks that are based on spike summation generated by neurons of Hodgkin–Huxley type or it’s derivations), is that even for relatively weak coupling the synchronization of some modes in network (11) and (12) may happen in a very efficient manner. The conditions for coupling coefficients when this synchronized state is realized and every mode *i* of the network produce the same activity pattern as sensory excited single mode, but without any external excitation, can be expressed for every mode *i* as

26 $$\begin{aligned} \sum _{j} w_{ij}\cos (\delta _{ij})&= \gamma _0, \end{aligned}$$

27 $$\begin{aligned} \sum _{j} w_{ij}\sin (\delta _{ij})&= 0. \end{aligned}$$

This is necessary (but not sufficient) condition that shows that every recurrent path through the network, that is every brain wave loop that do not introduce nonzero phase delays, should generate the same level of amplitude excitation.

**Figure 2:**
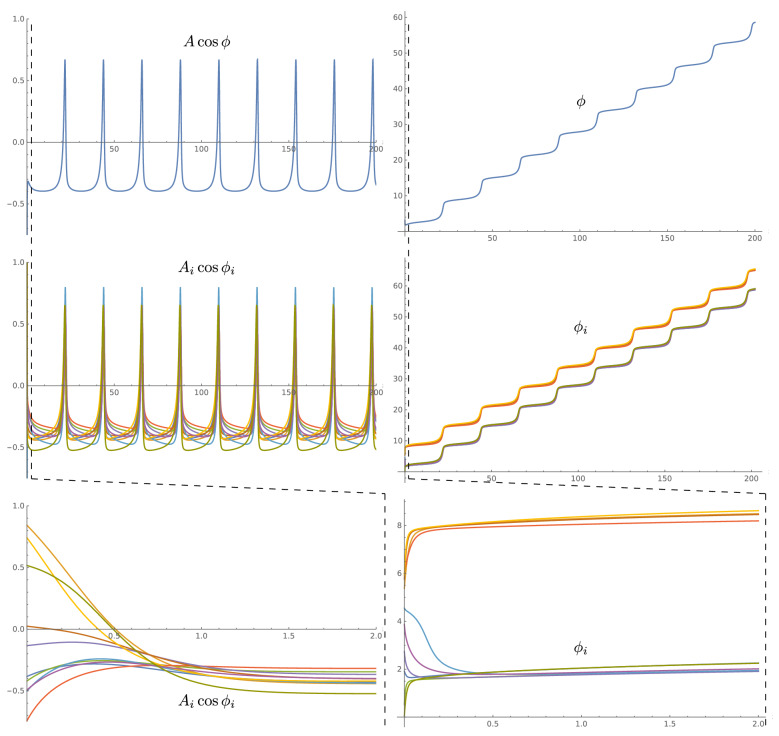
(Top) The amplitude and phase of a single mode subcritical spiking. (Middle) The spiking of multiple modes with different linear frequencies $\omega _i$ critically synchronized at the same effective spiking frequency (the units are arbitrary). The details of wavefront shapes for each mode are different, but the spiking synchronization between modes is very strong and precise. (Bottom) Expanded view of the initial part of the amplitude and phase of the mode shows the efficiency of synchronization—synchronization happens even faster than the single period of linear oscillations.

Even for this already oversimplified case of identical parameters, the currently agreed lines of research proceed with even more simplifications and either employ constant (small) amplitude phase synchronization approach (Kuramoto oscillators) assuming that all $\delta _{ij}$ equal to $-\pi /2$ or $\pi /2$, or use amplitude coupling (Hodgkin–Huxley neuron and the like) with $\delta _{ij}$ equal to 0 (excitatory) or $\pi$ (inhibitory). Both of these cases are extremely limited and do not provide a framework for the effectiveness, flexibility, adaptability, and robustness characteristic of human brain functioning. The phase coupling is only capable of generating very slow and inefficient synchronization. The amplitude coupling is even less efficient as it completely ignores the details of the phase of the incoming signal, thus is only able to produce sporadic and inconsistent population level synchronization.

**Figure 3:**
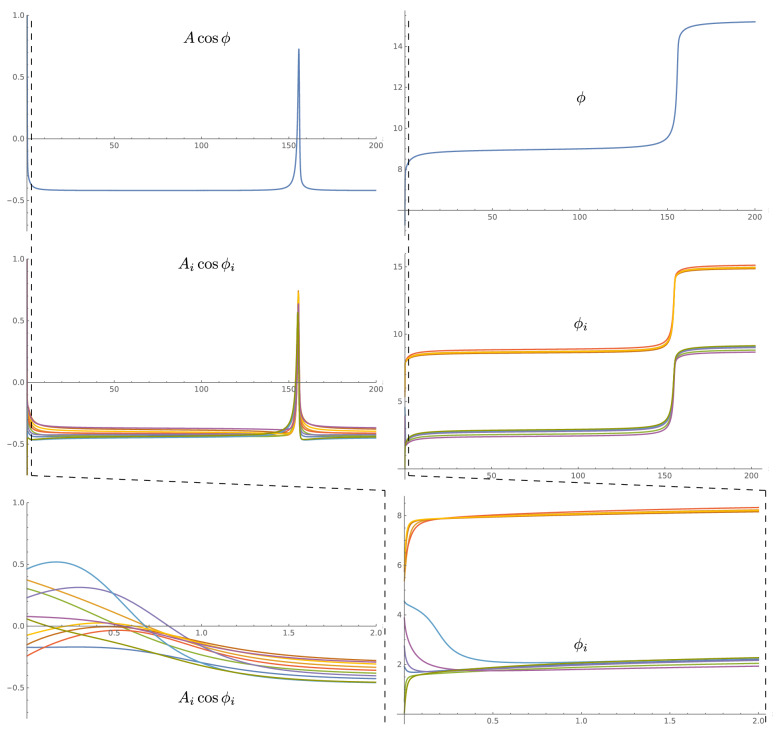
(Top) The amplitude and phase of a single mode spiking in a close to critical regime. (Middle) The spiking of multiple modes with different linear frequencies $\omega _i$ critically synchronized at the same effective spiking frequency that is close to critical frequency (the units are arbitrary). Similar to subcritical spiking in Fig. 2, the details of wavefront shapes for each mode are different, but the spiking synchronization between modes is very strong and precise. (Bottom) Expanded view of the initial part of the amplitude and phase of the mode shows the efficiency of synchronization–synchronization happens.

Of course, (26) and (27) are used as an idealized illustrative picture of critically synchronized memory state formation in phase–amplitude coupled network (11) and (12). In practice, in the brain the parameters of network (11) and (12), including frequencies, excitations, and other parameters of a single mode Hamiltonian (6), may be different between modes. But even in this case the formation of critically synchronized state follows the same outlined above procedure, and requires that for all modes total inputs to the phase and the amplitude parts (${\bar{\omega }}_i$ and ${\bar{\gamma }}_i$) generate together the same effective frequency $\omega _s$ satisfying the relation

28 $$\begin{aligned} \omega _s = \sqrt{{\bar{\omega }}_i^2 - {\bar{\gamma }}_i^2 w_i^2}, \end{aligned}$$

where

29 $$\begin{aligned} {\bar{\gamma }}_i&= \gamma _i + \sum _{j} w_{ij}\cos (\delta _{ij}), \end{aligned}$$

30 $$\begin{aligned} {\bar{\omega }}_i&= \omega _i + \sum _{j} w_{ij}\sin (\delta _{ij}). \end{aligned}$$

Overall, the critically synchronized memory can be formed by making a loop from as few as two modes. Of course, this may require too large an amount of amplitude coupling and will not produce the flexibility and robustness of multimode coupling with smaller steps of adjustment of amplitude–phase coupling parameters. Figures 2 and 3 show two examples of network synchronization with effective frequencies that replicate the original single mode effective frequency without sensory input. Ten modes were shown with the same parameters of $w_i^a = w_i^\phi = \sqrt{5}$, $\psi _i = 2\arctan {(1/3)}$, $\phi _i^c =\arctan {(1/2)}$, $w_i = 1/2$ but with a set of uniformly distributed frequencies $\omega _i$, (with a mean of 1 and a standard deviation of 0.58–0.59). The network coupling $w_{ij}$ and $\delta _{ij}$ were also selected from a range of values (from 0 to 0.2 for $w_{ij}$ and $-\pi /2$ to $\pi /2$ for $\delta _{ij}$).

**Figure 4:**
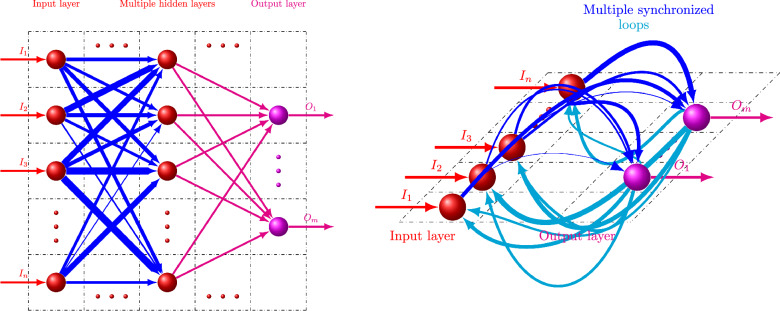
(Left) Schematic graph of typical multi-layer neural network where connection weights are shown by varying path width. (Right) Schematic graph of critically synchronized WETCOW inspired shallow neural network, where amplitude weighting factors are again shown by varying path widths, but an additional phase parameter controls the non-planar recurrent paths behavior. The interplay of amplitude-phase synchronization, shown by a non-planarity of a shallow—comprised of a single layer of synchronized loops—neural network, allows more efficient computations, memory requirements, and learning capabilities than multi-layer deep ARCSes of traditional AI/ML neural networks.

Again, for phase only coupling ($\delta _{ij}$ equal to $-\pi /2$ or $\pi /2$) the synchronization is very inefficient and only happening as a result of an emergence of forced oscillations at common frequency in some parts of the network or in the whole network dependent on the details of the coupling parameters. The amplitude coupling of Hodgkin–Huxley and the like neurons is even less effective than phase-only coupling as it does not even consider the oscillatory and wave-like propagation nature of the subthreshold signals that contribute to the input and collective generation of spiking output. Therefore, expressions (28) to (30) are not applicable for HH and LIF models as phase information, as well as frequency dependence, is lost by those models and replaced by ad-hoc sets of thresholds and time constants.

Contrary to the lack of efficiency, flexibility, and robustness demonstrated by those state-of-the-art curtailed phase-only and amplitude-only approaches, the presented model of memory shows that when both phase and amplitude are operating together, a critical behavior emerging in the nonlinear system (9) gives birth to an efficient, flexible, and robust synchronization characteristic of human memory, appropriate for any type of coding, being it either rate or time.

## Application to neural networks and machine learning

The presented critically synchronized memory model based on the theory of weakly evanescent brain waves—WETCOW^60–62^—has several very important properties. First of all, the presence of both amplitude $w_{ij}$ and phase $\delta _{ij}$ coupling makes if possible to construct an effective and accurate recurrent networks that do not require extensive and time consuming training. The standard back propagation approach can be very expensive in terms of both computations, memory requirements, and large amount of communications involved, therefore may be poorly suited to the hardware constraints in computers and neuromorphic devices^78^. However, with the WETCOW based model it is easy to construct a small shallow network that will replicate the spiking produced by any input condition using the interplay of the amplitude–phase coupling (19) to (20) and the explicit analytical conditions for spiking rate (13) and (15) as a function of criticality. The shallow neural networks constructed using those analytical conditions give very accurate results with very little amount of training and very little memory requirements.

Comparison of a schematic diagram for the typical workflow of traditional multi-layer ARCSe neural network with a diagram for the critically synchronized WETCOW inspired shallow neural network is shown in Fig. 4. For the traditional multi-layer ARCSe neural network the diagram (Left panel) includes the connection weights that are approximated by varying path width. The Schematic diagram for the critically synchronized WETCOW inspired shallow neural network in addition to amplitude weighting factors (that again shown by varying recurrent path widths) has an additional phase parameter that is shown by the non-planarity of the connection paths. The presence of non-planarity in a single layer (shallow) amplitude-phase synchronized neural network allows more efficient computations, memory requirements, and learning capabilities than multi-layer deep ARCSes of traditional AI/ML neural networks.

The non-planarity of the critically synchronized WETCOW inspired shallow neural network shown in Fig. 4 illustrates and emphasizes another important advantage in comparison to the traditional multi-layer ARCSe neural networks. It is well known that the traditional multi-layer deep learning ARCSe neural network models suffer from the phenomenon of catastrophic forgetting—a deep ARCSe neural network, carefully trained and back and forth massaged to perform some important task can unexpectedly lose its generalization ability on this task after additional training on a new task has been performed^79–81^. This typically happens because a new task overrides the previous weights that have been learned in the past. This means that continues learning degrades or even destroys the model performance for the previous tasks. This is a tremendous problem meaning that the traditional deep ARCSe neural network represents a very bad choice to function as a continuous learner, as it constantly forgets the previously learned knowledge being exposed to a bombardment of a new information. As any new information added to the traditional multi-layer deep learning ARCSe neural network inevitably modifies the network weights confined to the same plane and shared with all previously accumulated knowledge produced by a hard training work, this catastrophic forgetting phenomena is generally not a surprise. The non-planarity of the critically synchronized WETCOW inspired shallow neural network provides an additional way to encode new knowledge with a different out-of-plane phase-amplitude choice, thus preserving previous accumulated knowledge. This makes the critically synchronized WETCOW inspired shallow neural network model more suitable for the use in a continuous learning scenario.

Another important advantage of the WETCOW algorithms is their numerical stability, which makes them robust even in the face of extensive training. Because the system (19) and (20) describes the full range of dynamics, from linear oscillations to spiking in the perfectly differentiable form, it is perfectly differentiable. They thus are not subject to one of the major limitations of current standard models—the non-differentiability of the spiking nonlinearity for LIF (and similar) models, whose derivative is zero everywhere except at $U = \Theta$, and even at $U=\Theta$ the derivatives is not just large, but strictly speaking they are not defined.

## MNIST digits and MNIST fashion tests

**Figure 5:**
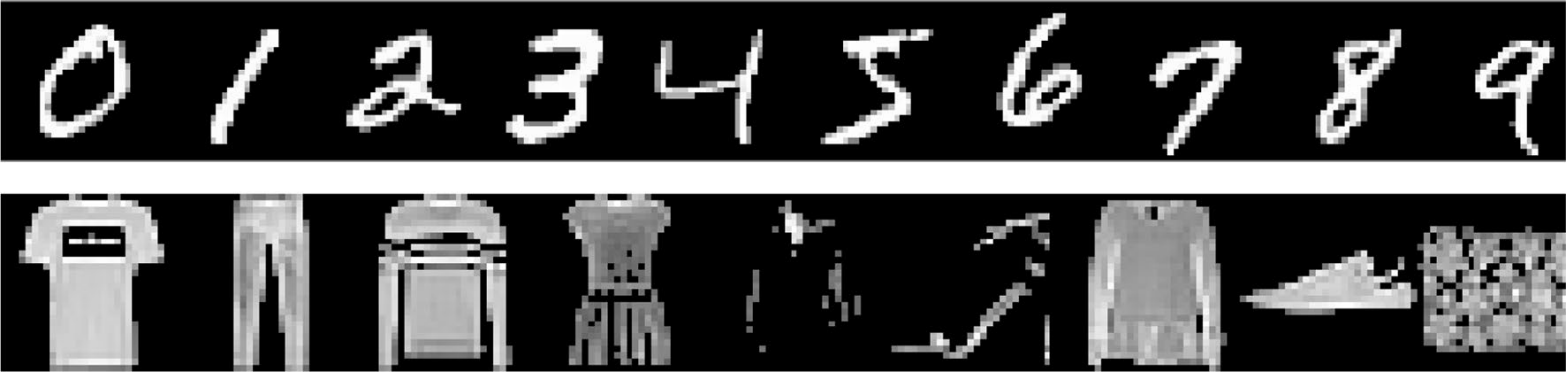
(Top) Several example images from MNIST database of handwritten digits. (Bottom) Several example images from MNIST-like fashion product database of Zalando’s article images designed as a direct drop-in replacement for the original MNIST dataset.

The performance and accuracy of WETCOW based learning approaches is easily demonstrated on two commonly used databases: MNIST^82^ and Fashion-MNIST^83^. Both the original handwritten digits MNIST database Fig. 5 (top) and an MNIST-like fashion product database—dataset of Zalando’s article images designed as a direct drop-in replacement for the original MNIST dataset—Fig. 5 (bottom) contain 60,000 training images and 10,000 testing images. Each individual image is a $28\times 28$ pixels grayscale image, associated with a single label from 10 different label classes.

The results for our WETCOW based model for a shallow recurrent neural network applied to the MNIST handwritten digits Fig. 5 (top) and MNIST fashion images Fig. 5 (bottom) are summarized in Table 1. In both cases the networks were generated for $7\times 7$ downsampled images moved and rescaled to the common reference system. For each of the datasets, Table 1 shows two entries, the first corresponds to an initial construction of a recurrent network that involves just a single iteration, without any back propagation and retraining steps. In both cases this initial step produces very good initial accuracy, on par or even exceeding the final results of some of the deep ARCSes^84, 85^. The second entry for each dataset shows highest accuracy achieved and the corresponding training times. Both entries confirm that to achieve the accuracies that are higher than the accuracies obtained by any of the deep ARCSes orders of magnitude smaller training times are required.

**Table 1:** Summary of accuracy and timing results obtained by shallow learning processing of the original handwritten digits MNIST dataset^82^ and the Fashion-MNIST dataset^83^ using WETCOW inspired algorithm and test implementation based on the ideas of critically synchronized learning.

	Accuracy	Time	Others
MNISTDigits (without training)	0.9858–0.9883 (117–142 errors per 10,000 samples)	Several seconds	
MNISTDigits (with training)	0.9986 (14 errors per 10,000 samples)	Several minutes	0.88–0.998 14 h for 0.9977 accuracy
MNISTFashion (without training)	0.9385 (615 errors per 10,000 samples)	Several seconds	
MNISTFashion (with training)	0.9742 (258 errors per 10,000 samples)	Several minutes	0.444–0.897 From 1 to 50 h

## Conclusion

This paper presents arguments and test results showing that recently developed physics based theory of wave propagation in the cortex—the theory of weakly evanescent brain waves—WETCOW^60–62^—provides both a theoretical and computational framework with which to better understand the adaptivity, flexibility, robustness, and effectiveness of human memory, and, hence, can be instrumental in development of novel learning algorithms. Those novel algorithms potentially allow the achievement of extreme data efficiency and adaptive resilience in dynamic environments, characteristic of biological organisms. The test examples based on our WETCOW inspired algorithms show excellent performance (orders of magnitude faster than current state-of-the-art deep ARCSe methods) and accuracy (exceeding the accuracy of current state-of-the-art deep ARCSe methods) and can be expected to be resilient to catastrophic forgetting, and will demonstrate real-time sensing, learning, decision making, and prediction. Due to very efficient, fast, robust and very precise spike synchronization, the WETCOW based algorithms are able to respond to novel, uncertain, and rapidly changing conditions in real-time, and will enable well-informed decisions based on small amounts of data over short time horizons. The WETCOW based algorithms can include uncertainty quantification for data of high sparsity, large size, mixed modalities, and diverse distributions, and will push the bounds on out-of-distribution generalization.

The paper presents ideas of how to extract principles, not available from current neural network approaches, by which biological learning occurs through wave dynamic processes arising in neuroanatomical structures, and in turn provides a new framework for the design and implementation of highly efficient and accurate engineering analogs of those processes and structures that could be instrumental in the design of novel learning circuits.

## Figures and Tables

**Figure 6:**
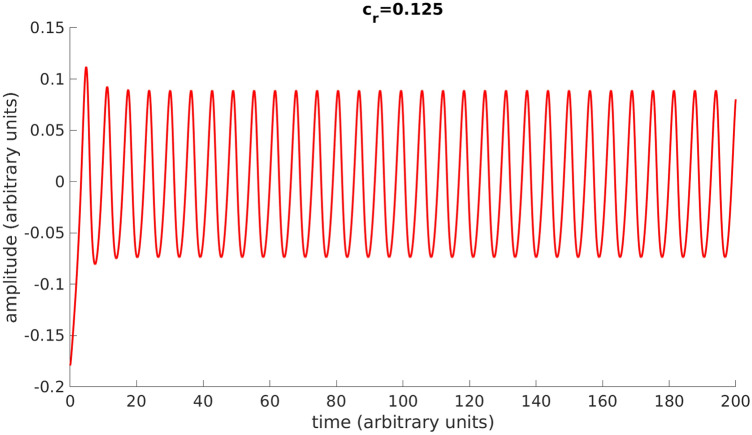
The amplitude and phase of a single mode for $c_r=0.125$.

**Figure 7:**
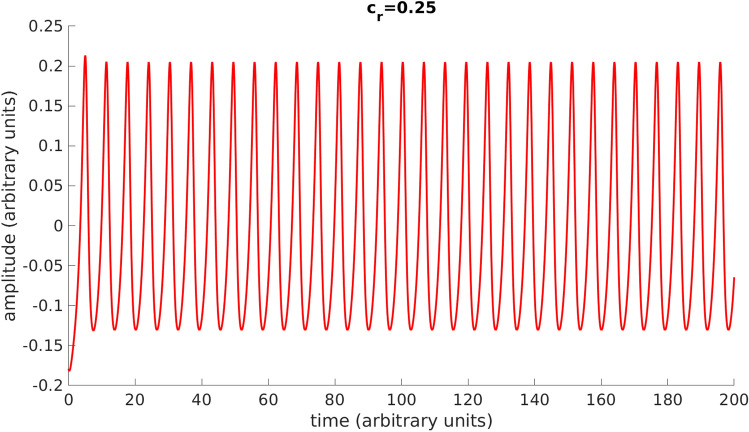
The amplitude and phase of a single mode for $c_r=0.25$.

**Figure 8:**
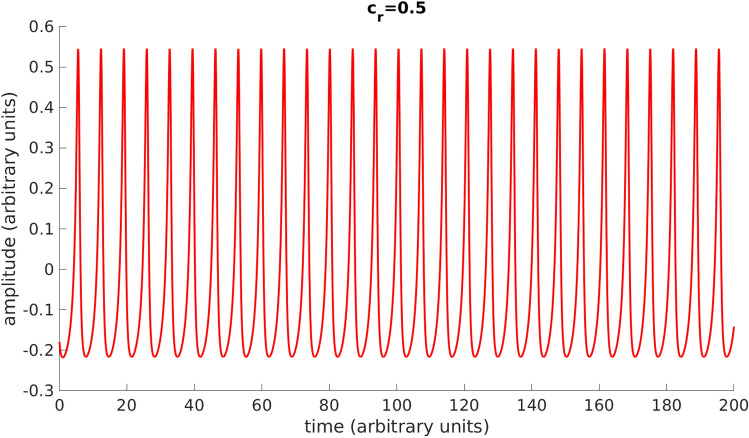
The amplitude and phase of a single mode for $c_r=0.5$.

**Figure 9:**
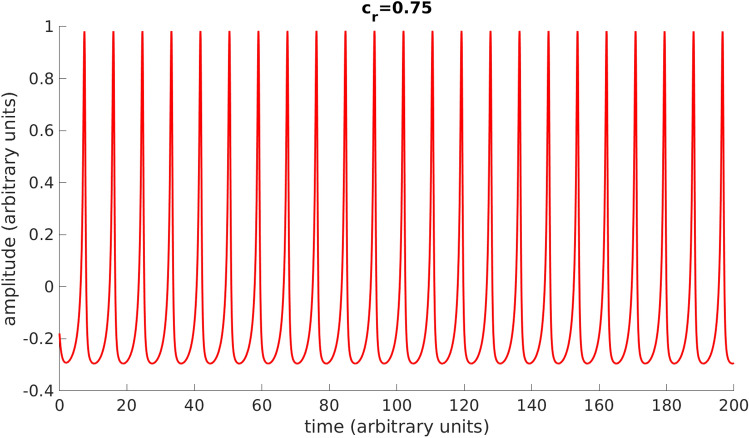
The amplitude and phase of a single mode for $c_r=0.75$.

**Figure 10:**
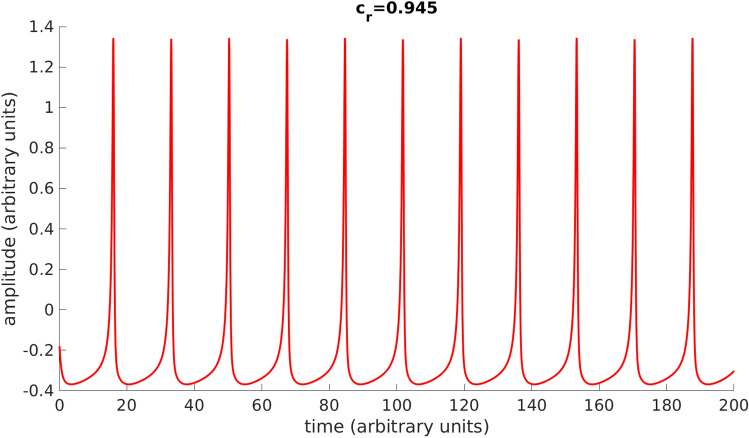
The amplitude and phase of a single mode for $c_r=0.945$.

**Figure 11:**
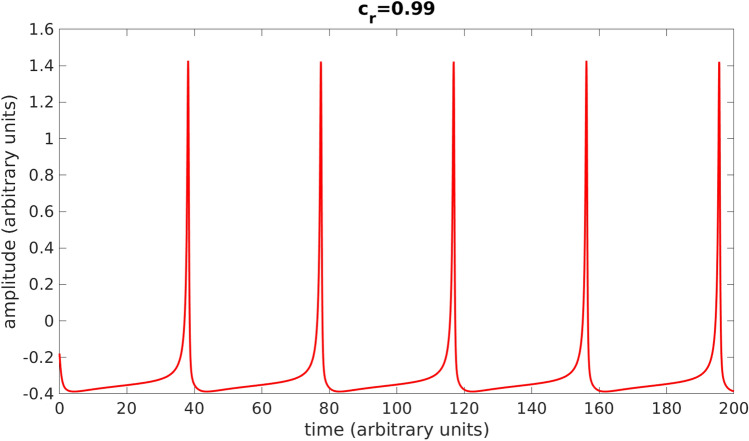
The amplitude and phase of a single mode for $c_r=0.99$.

**Figure 12:**
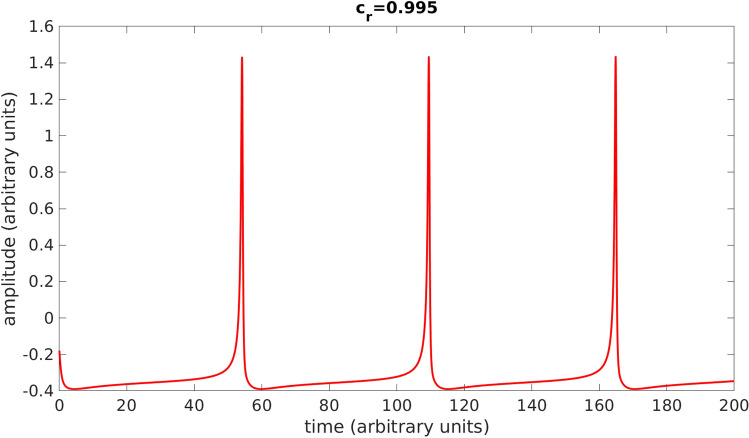
The amplitude and phase of a single mode for $c_r=0.995$.

**Figure 13:**
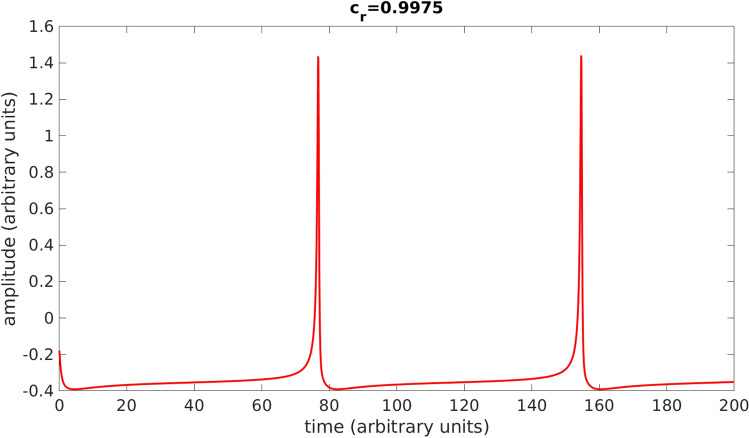
The amplitude and phase of a single mode for $c_r=0.9975$.

## Data Availability

The datasets used and/or analyzed during the current study available from the corresponding author on reasonable request.

## Appendix: Several examples of single mode solutions

See Figs. 6, 7, 8, 9, 10, 11, 12 and 13.

## References

[1] Hodgkin AL, Huxley AF (1952). A quantitative description of membrane current and its application to conduction and excitation in nerve. J. Physiol. (Lond.).

[2] Johnston D, Wu SM-S (1994). Foundations of Cellular Neurophysiology (A Bradford Book).

[3] Giannari A, Astolfi A (2022). Model design for networks of heterogeneous Hodgkin–Huxley neurons. Neurocomputing.

[4] Strassberg AF, DeFelice LJ (1993). Limitations of the Hodgkin–Huxley formalism: Effects of single channel kinetics on transmembrane voltage dynamics. Neural Comput..

[5] Meunier C, Segev I (2002). Playing the devil’s advocate: Is the Hodgkin–Huxley model useful?. Trends Neurosci..

[6] Yamazaki K, Vo-Ho V-K, Bulsara D, Le N (2022). Spiking neural networks and their applications: A review. Brain Sci..

[7] Fitzhugh R (1961). Impulses and physiological states in theoretical models of nerve membrane. Biophys. J..

[8] Nagumo J, Arimoto S, Yoshizawa S (1962). An active pulse transmission line simulating nerve axon. Proc. IRE.

[9] Morris C, Lecar H (1981). Voltage oscillations in the barnacle giant muscle fiber. Biophys. J..

[10] Izhikevich EM (2003). Simple model of spiking neurons. IEEE Trans. Neural Netw..

[11] Zenke F, Ganguli S (2018). SuperSpike: Supervised learning in multilayer spiking neural networks. Neural Comput..

[12] Kaiser J, Mostafa H, Neftci E (2020). Synaptic plasticity dynamics for deep continuous local learning (DECOLLE). Front. Neurosci..

[13] Tavanaei A, Ghodrati M, Kheradpisheh SR, Masquelier T, Maida A (2019). Deep learning in spiking neural networks. Neural Netw..

[14] Bellec G (2020). A solution to the learning dilemma for recurrent networks of spiking neurons. Nat. Commun..

[15] Hunsberger, E. & Eliasmith, C. Training spiking deep networks for neuromorphic hardware. arXiv:1611.05141. 10.13140/RG.2.2.10967.06566 (2016).

[16] Sengupta A, Ye Y, Wang R, Liu C, Roy K (2019). Going deeper in spiking neural networks: VGG and residual architectures. Front. Neurosci..

[17] Rueckauer B, Lungu I-A, Hu Y, Pfeiffer M, Liu S-C (2017). Conversion of continuous-valued deep networks to efficient event-driven networks for image classification. Front. Neurosci..

[18] Xu Q (2022). Hierarchical spiking-based model for efficient image classification with enhanced feature extraction and encoding. IEEE Trans. Neural Netw. Learn. Syst..

[19] Shen J, Zhao Y, Liu JK, Wang Y (2021). HybridSNN: Combining bio-machine strengths by boosting adaptive spiking neural networks. IEEE Trans. Neural Netw. Learn. Syst..

[20] Kheradpisheh SR, Ganjtabesh M, Thorpe SJ, Masquelier T (2018). Stdp-based spiking deep convolutional neural networks for object recognition. Neural Netw..

[21] Kim S, Park S, Na B, Yoon S (2020). Spiking-yolo: Spiking neural network for energy-efficient object detection. Proc. AAAI Conf. Artif. Intell..

[22] Zhou S, Chen Y, Li X, Sanyal A (2020). Deep scnn-based real-time object detection for self-driving vehicles using lidar temporal data. IEEE Access.

[23] Luo, Y. *et al.* Siamsnn: Spike-based siamese network for energy-efficient and real-time object tracking. arXiv:2003.07584 (arXiv preprint) (2020).

[24] Bertinetto, L., Valmadre, J., Henriques, J. F., Vedaldi, A. & Torr, P. H. Fully-convolutional siamese networks for object tracking. In *Computer Vision–ECCV 2016 Workshops: Amsterdam, The Netherlands, October 8–10 and 15–16, 2016, Proceedings, Part II 14*, 850–865 (Springer, 2016).

[25] Patel, K., Hunsberger, E., Batir, S. & Eliasmith, C. A spiking neural network for image segmentation. arXiv:2106.08921 (arXiv preprint) (2021).

[26] Rasmussen D (2019). Nengodl: Combining deep learning and neuromorphic modelling methods. Neuroinformatics.

[27] Rostami A, Vogginger B, Yan Y, Mayr CG (2022). E-prop on SpiNNaker 2: Exploring online learning in spiking RNNs on neuromorphic hardware. Front. Neurosci..

[28] Muller Cleve SF (2022). Braille letter reading: A benchmark for spatio-temporal pattern recognition on neuromorphic hardware. Front. Neurosci..

[29] Lee ST, Bae JH (2022). Investigation of deep spiking neural networks utilizing gated Schottky diode as synaptic devices. Micromachines (Basel).

[30] Paul A, Tajin MAS, Das A, Mongan WM, Dandekar KR (2022). Energy-efficient respiratory anomaly detection in premature newborn infants. Electronics (Basel).

[31] Petschenig H (2022). Classification of Whisker deflections from evoked responses in the somatosensory barrel cortex with spiking neural networks. Front. Neurosci..

[32] Patino-Saucedo A, Rostro-Gonzalez H, Serrano-Gotarredona T, Linares-Barranco B (2022). Liquid state machine on SpiNNaker for spatio-temporal classification tasks. Front. Neurosci..

[33] Li K, Ncipe JC (2021). Biologically-inspired pulse signal processing for intelligence at the edge. Front. Artif. Intell..

[34] Syed T, Kakani V, Cui X, Kim H (2021). Exploring optimized spiking neural network architectures for classification tasks on embedded platforms. Sensors (Basel).

[35] Fil J, Chu D (2020). Minimal spiking neuron for solving multilabel classification tasks. Neural Comput..

[36] Saucedo A, Rostro-Gonzalez H, Serrano-Gotarredona T, Linares-Barranco B (2020). Event-driven implementation of deep spiking convolutional neural networks for supervised classification using the SpiNNaker neuromorphic platform. Neural Netw..

[37] Liu G, Deng W, Xie X, Huang L, Tang H (2022). Human-level control through directly trained deep spiking Q-networks. IEEE Trans. Cybern..

[38] Zhang M (2022). Rectified linear postsynaptic potential function for backpropagation in deep spiking neural networks. IEEE Trans. Neural Netw. Learn. Syst..

[39] Kwon D (2020). On-chip training spiking neural networks using approximated backpropagation with analog synaptic devices. Front. Neurosci..

[40] Lee J, Zhang R, Zhang W, Liu Y, Li P (2020). Spike-train level direct feedback alignment: Sidestepping backpropagation for on-chip training of spiking neural nets. Front. Neurosci..

[41] Meng Q (2022). Training much deeper spiking neural networks with a small number of time-steps. Neural. Netw..

[42] Chen Y, Du J, Liu Q, Zhang L, Zeng Y (2019). Robust and energy-efficient expression recognition based on improved deep ResNets. Biomed. Tech. (Berl.).

[43] Pfeiffer M, Pfeil T (2018). Deep learning with spiking neurons: Opportunities and challenges. Front. Neurosci..

[44] Stromatias E (2015). Robustness of spiking Deep Belief Networks to noise and reduced bit precision of neuro-inspired hardware platforms. Front. Neurosci..

[45] Thiele JC, Bichler O, Dupret A (2018). Event-based, timescale invariant unsupervised online deep learning with STDP. Front. Comput. Neurosci..

[46] Neftci E, Das S, Pedroni B, Kreutz-Delgado K, Cauwenberghs G (2013). Event-driven contrastive divergence for spiking neuromorphic systems. Front. Neurosci..

[47] Kim Y, Panda P (2021). Revisiting batch normalization for training low-latency deep spiking neural networks from scratch. Front. Neurosci..

[48] Zou C (2021). A scatter-and-gather spiking convolutional neural network on a reconfigurable neuromorphic hardware. Front. Neurosci..

[49] Liu F (2021). SSTDP: Supervised spike timing dependent plasticity for efficient spiking neural network training. Front Neurosci.

[50] Zhang L (2021). A cost-efficient high-speed VLSI architecture for spiking convolutional neural network inference using time-step binary spike maps. Sensors (Basel).

[51] Zhan Q, Liu G, Xie X, Sun G, Tang H (2022). Effective transfer learning algorithm in spiking neural networks. IEEE Trans. Cybern..

[52] Detorakis G (2018). Neural and synaptic array transceiver: A brain-inspired computing framework for embedded learning. Front. Neurosci..

[53] Guo W, Fouda ME, Yantir HE, Eltawil AM, Salama KN (2020). Unsupervised adaptive weight pruning for energy-efficient neuromorphic systems. Front. Neurosci..

[54] Miranda EJ (2020). Memristors for neuromorphic circuits and artificial intelligence applications. Materials (Basel).

[55] Gale EM (2019). Neuromorphic computation with spiking memristors: Habituation, experimental instantiation of logic gates and a novel sequence-sensitive perceptron model. Faraday Discuss.

[56] Esser SK (2016). Convolutional networks for fast, energy-efficient neuromorphic computing. Proc. Natl. Acad. Sci. USA.

[57] Davidson S, Furber SB (2021). Comparison of artificial and spiking neural networks on digital hardware. Front. Neurosci..

[58] Gerstner W, Kistler WM, Naud R, Paninski L (2014). Neuronal dynamics: From single neurons to networks and models of cognition.

[59] Sardi S, Vardi R, Sheinin A, Goldental A, Kanter I (2017). New types of experiments reveal that a neuron functions as multiple independent threshold units. Sci. Rep..

[60] Galinsky VL, Frank LR (2020). Universal theory of brain waves: From linear loops to nonlinear synchronized spiking and collective brain rhythms. Phys. Rev. Res..

[61] Galinsky VL, Frank LR (2020). Brain waves: Emergence of localized, persistent, weakly evanescent cortical loops. J. Cogni. Neurosci..

[62] Galinsky VL, Frank LR (2021). Collective synchronous spiking in a brain network of coupled nonlinear oscillators. Phys. Rev. Lett..

[63] Seth AK, Bayne T (2022). Theories of consciousness. Nat. Rev. Neurosci..

[64] Humeau Y, Choquet D (2019). The next generation of approaches to investigate the link between synaptic plasticity and learning. Nat. Neurosci..

[65] Miller SM, Sahay A (2019). Functions of adult-born neurons in hippocampal memory interference and indexing. Nat. Neurosci..

[66] Feng S, Duarte MF (2019). Few-shot learning-based human activity recognition. Expert Syst. Appl..

[67] Wang Y, Yao Q, Kwok JT, Ni LM (2020). Generalizing from a few examples. ACM Comput. Surv..

[68] Drori I (2022). A neural network solves, explains, and generates university math problems by program synthesis and few-shot learning at human level. Proc. Natl. Acad. Sci..

[69] Walsh R, Abdelpakey MH, Shehata MS, Mohamed MM (2022). Automated human cell classification in sparse datasets using few-shot learning. Sci. Rep..

[70] Galinsky VL, Frank LR (2023). Critical brain wave dynamics of neuronal avalanches. Front. Phys..

[71] Galinsky VL, Frank LR (2023). Neuronal avalanches: Sandpiles of self organized criticality or critical dynamics of brain waves?. Front. Phys..

[72] Galinsky, V. L. & Frank, L. R. Neuronal avalanches and critical dynamics of brain waves. arXiv:2111.07479 (eprint) (2021). 10.1007/s11467-023-1273-7 PMC10062440 37008280

[73] Kuramoto Y, Araki H (1975). Self-entrainment of a population of coupled non-linear oscillators. Mathematical Problems in Theoretical Physics, vol 39 of Lecture Notes in Physics.

[74] Kuramoto Y, Battogtokh D (2002). Coexistence of coherence and incoherence in nonlocally coupled phase oscillators. Nonlinear Phenom. Complex Syst..

[75] Kuramoto Y, Hogan J (2002). Reduction methods applied to non-locally coupled oscillator systems. Nonlinear Dynamics and Chaos: Where do We Go from Here?.

[76] Kulkarni A, Ranft J, Hakim V (2020). Synchronization, stochasticity, and phase waves in neuronal networks with spatially-structured connectivity. Front. Comput. Neurosci..

[77] Kim R, Sejnowski TJ (2021). Strong inhibitory signaling underlies stable temporal dynamics and working memory in spiking neural networks. Nat. Neurosci..

[78] Neftci EO, Mostafa H, Zenke F (2019). Surrogate gradient learning in spiking neural networks: Bringing the power of gradient-based optimization to spiking neural networks. IEEE Signal Process. Mag..

[79] Kirkpatrick J (2017). Overcoming catastrophic forgetting in neural networks. Proc. Natl. Acad. Sci. USA.

[80] Kemker, R., McClure, M., Abitino, A., Hayes, T. & Kanan, C. Measuring catastrophic forgetting in neural networks (2017). arXiv:1708.02072.

[81] Ramasesh, V. V., Dyer, E. & Raghu, M. Anatomy of catastrophic forgetting: Hidden representations and task semantics (2020). arXiv:2007.07400.

[82] LeCun, Y., Cortes, C. & Burges, C. Mnist handwritten digit database. *ATT Labs [Online]*. http://yann.lecun.com/exdb/mnist **2** (2010).

[83] Xiao, H., Rasul, K. & Vollgraf, R. Fashion-mnist: A novel image dataset for benchmarking machine learning algorithms (2017). arXiv:1708.07747 [cs.LG].

[84] Xiao, H., Rasul, K. & Vollgraf, R. A mnist-like fashion product database. Benchmark—Github. https://github.com/zalandoresearch/fashion-mnist (2022). Accessed 21 Ma 2022.

[85] MNIST database. Mnist database—Wikipedia, the free encyclopedia (2022). Accessed 31 July 2022.

